# The Treatment of Typhoid Fever

**Published:** 1892-12-10

**Authors:** 


### THE HOSPITAL CLINIC.

[*The Editor will be glad to receive offers of co-operation and contributions from members of the profession. All letters should be addressed to* THE EDITOR, THE LODGE, PORCHESTER SQUARE, LONDON, W.]

### LIVERPOOL ROYAL INFIRMARY.

### THE TREATMENT OF TYPHOID FEVER.

When a patient suspected to be suffering from typhoid fever is sent to the wards, he is put to bed at once without receiving the usual preliminary bath, and is washed as far as needful afterwards. If the suspicion of typhoid fever is confirmed, the patient is maintained in the recumbent position throughout the illness, and is on no account permitted to sit up, or to make any exertion until convalescence is established. Light bedclothes only are allowed, and the ward is kept at a temperature of from 60 to 65 deg. Fahr. For diet four ounces of milk diluted with half the quantity of lime-water are given every two hours night and day, and in many cases the milk is previously boiled. Every evening the patient is cautiously sponged over with tepid water, and if sordes form on the lips and teeth they are from time to time removed by the application of glycerine and borax, or better still, glycerine mixed with an equal amount of the juice of a lemon. If the bowels act, some carbolic lotion (one in twenty) is placed in the bed-pan, and the evacuations are kept for the physician to see. The restricted dietary is adhered to for at least a week after the temperature has become normal, and no meat is allowed until it has been normal for three weeks.

Such a mode of treatment is manifestly in conformity with all we know of the pathology of the disease, and when carefully and steadily carried out, will suffice to lead most cases to a successful issue. Of late years, however, some of the physicians at the infirmary have supplemented this purely expectant treatment by the exhibition of antiseptics, with a view to the limitation or prevention of putrefactive changes in the intestines, and the consequent absorption of pyrogenic materials from the ulcers. The results hitherto obtained from this treatment have been very encouraging; under its use the mortality has been reduced, the average duration of the cases has been shortened, and the general course of the disease has been much milder. Some striking results of it have been brought forward by Dr. Caton in a paper in the *British Medical Journal* of July 23rd, 1892, where the progress and result of 44 cases of typhoid fever are reported, together with the line of treatment adopted in each case. In half the cases intestinal antiseptics were given; in the other half they were not given. All the cases treated by antiseptics recovered; of the others four died. The relative severity of the two sets of cases may be gathered from the subjoined table, which shows the number of deaths and the average duration of fever, and of relapses, and the average stay in the hospital of the cases that got well:—

| Treatment. | Cases. | Deaths. | Days of Fever. | Days of Relapse. | Days in Hosptl. |
|---|---|---|---|---|---|
| Expectant (or with quinine) | 22 | 4 | 37·9 | 9 | 52 |
| Intestinal antisepsis | 22 | 0 | 25·3 | 1·8 | 46 |

Among antiseptics, Dr. Caton prefers naphthalin in the form of a pill, and gives daily six or eight pills containing three or four grains of the drug in each.

Excellent results have lately been obtained in the infirmary from the powders recommended by Professor Bouchard, which consist of 5 grains B-naphthol and 2½ grains of salicylate of bismuth. One of these is given with the milk every two, four, or six hours, according to the severity of the case. If the taste is objected to, they may be given in wafer paper, the paper being dipped in water and then wrapped around the powder. Theoretically, the maximum effect of this line of treatment is reached when the motions become free from smell, but excellent results are obtained short of pushing it so far. The rapid improvement as regards the strength, temperature, and general condition under this treatment has been often very striking.

As a general rule, milk alone forms sufficient dietary, and patients seldom fail to take it well, though they may complain of its monotony. Some physicians order beef tea, and others allow a cup of tea freshly drawn; the latter does not appear to be contra-indicated in this disease, and may be beneficial from its stimulating and diuretic action.

Alcoholic stimulants are, as a rule, given at some period of the illness. The rate and quality of the pulse form the main guide for their administration. So long as the pulse remains strong and of moderate rate, say, below 100, they are usually withheld. A weak, thready pulse, especially if it be dicrotic, calls for their administration. A small amount—3 oz. of brandy or 4 oz. of port wine—is usually first employed, and the effect carefully watched. If the pulse becomes quicker, and especially if the face becomes hot and flushed, they are discontinued; if no evil effects appear the amount is increased in proportion to the gravity of the symptoms. It is not often found necessary to give more than 6 oz. of brandy in the 24 hours, or a proportionate amount of port wine. Whatever stimulant is employed is given in divided doses, well diluted, every two or three hours. Brandy may be given with the milk. For urgent adynamic cases the best stimulant is champagne, to which brandy may be added.

The more serious complications of typhoid fever happily do not seem to be so often met with since the antiseptic treatment has come into general use. One that not infrequently calls for interference is excessive rise of temperature. So long as it does not exceed 104 deg. Fahr., no special treatment is employed beyond the daily sponging above described. If it shows a tendency to rise sponging is more frequently resorted to, and if this fails to reduce it, the wet pack is employed. The pack is administered as follows: A waterproof sheet is slipped under the patient, and his sleeping clothes are removed. A sheet wrung out of tepid water until it is nearly dry is then wrapped round him so that only the face is left out, and so that the thermometer can be inserted into the rectum when required. A light, single blanket is thrown over the sheet. If the sheet dries quickly tepid water is sprinkled on it. The temperature must be taken every ten minutes in the mouth or rectum, and the pack left on until some reduction of temperature is effected. It is not desirable to wait for a reduction below 103 deg. or 102 deg., as the fall generally continues after the pack has been removed. A little brandy is given before it is applied. It probably helps the action of the pack by dilating the vessels of the skin. When the pack is removed the patient is quickly dried, dry clothing is supplied, and a hot water bottle placed at his feet. If no reduction of temperature takes place within an hour of applying the pack, it is probably useless to persevere with it, and it may then be necessary to place the patient in a bath. To do this, a full length bath is brought to the side of the bed and filled about one-half with water at 85 deg. A sheet may be laid across it, and the patient is lifted by three persons off the bed and lowered gently into the water. The temperature of the bath will tend to rise rapidly, and must be kept down by adding cold water or pieces of ice, while the hot water is withdrawn. Diarrhœa is not often found to require any special treatment, it is perhaps best controlled by enemata of starch and opium. Hæmorrhage is not of frequent occurrence.

An ice-bag would, in case of hæmorrhage be applied to the abdomen, and might be supplemented by a small enema of cold water. In the cases, not unfrequent, where constipation persists throughout the illness, the bowels are interfered with as little as possible. So long as no special uneasiness is present they are not interfered with as a rule; but if tympanites, or other evidence of fœcal accumulation, is present, it is relieved by an occasional simple enema, to which some tincture of assafœtida may be added. A glycerine injection is sometimes employed. Great watchfulness is maintained during convalescence, and ordinary food is returned to very gradually.

